# Odour characterisation of recycled HDPE in different washing and processing processes

**DOI:** 10.1007/s11356-024-34976-2

**Published:** 2024-09-23

**Authors:** Juan López Martínez, Jesús Manuel Rodríguez Rego, Laura Mendoza Cerezo, María Dolores Samper Madrigal, Antonio Macías-García

**Affiliations:** 1 Department of Mechanical and Materials Engineering, Polytechnic University of Valencia, Paseo Viaducto 1, 03801 Alcoy, Alicante Spain; 2 Department of Graphic Expression, School of Industrial Engineering, Universidad de Extremadura, Avda. de Elvas S/N, 06006 Badajoz, Spain; 3 Department of Mechanical, Energy and Materials Engineering, Universidad de Extremadura, Avda. S/N, 06006 Badajoz, Spain

**Keywords:** High-density polyethylene, Recycling, Characterisation, Odour

## Abstract

The waste of polymeric materials in our society is increasing year after year, generating a serious pollution problem. One way to deal with this waste problem is to recycle and reuse these materials. This process of recovery of used plastic materials aims to minimise their impact on the environment and reduce the energy consumption required for the generation of new consumer products. Recycling companies that recover these plastic materials must take into account some aspects such as transparency and colour, cleanliness, size, odour and sorting. One of the major disadvantages in accepting these recycled materials in the production processes is their odour, which in some cases causes the rejection of materials with comparable mechanical characteristics. High-density polyethylene, HDPE, is one of the polymeric wastes generated in the packaging industry. The aim of this work is to eliminate the bad odour of HDPE from waste collection plants for application in the recovery and reuse industry. HDPE supplied by a recycling company was washed, characterised and processed, and the odour was analysed by gas chromatography at each stage and by olfactory panel. In view of the results, it was observed that the washing processes managed to reduce the odour. Likewise, the processing of this waste by extrusion and injection managed to further reduce this effect, even eliminating some of the components responsible for odour by treating the samples with acetone and then extruding and injecting these samples. These results have a direct application in the packaging industry with significant shares of recycled material.

## Introduction

Polymers are long chains of small repeating molecules, called monomers, which due to their properties have a wide variety of industrial, commercial, and domestic applications.

The consumption of polymeric materials in our society continues to increase year after year; however, this growth in the production of plastics is also reflected in the amount of waste that we end up generating, especially in industries that use this material to package their products (Geyer et al. 2017; PlasticsEurope 2017*. Plastics—The Facts 2017: An Analysis of European Plastics Production, Demand and Waste Data 2017* n.d.; Radusin et al. 2020).

The amount of plastic waste generated has become a global problem; this type of pollution knows no borders (Jambeck et al. 2015; Lebreton et al. 2017), so the approach to solving it must be global. Society is tackling this waste problem by treating and reusing these materials, moving from a linear economy to a circular economy (Antonopoulos et al. 2021).

A second use of these plastic materials is essential to establish a market for recycled products. For this, it is necessary to achieve similar qualities between virgin materials and products that incorporate a percentage of recycled material in their manufacture (*Plastics - the Facts 2020 • Plastics Europe* n.d.).

The recovery process of used plastic materials aims to minimise their impact on the environment and reduce the energy consumption required for the generation of new consumer products. Recycling companies that reprocess plastic materials must take into account some aspects that allow them to best control the various properties of the recycled materials, including mechanical properties and aspects such as transparency and colour, cleanliness, size, odour and sorting.

One of the major drawbacks to accept these recycled materials in the production processes is their odour, which in some cases causes the rejection of materials with mechanical characteristics comparable to those of the virgin material, but which, due to the presence of this characteristic bad odour, makes it impossible to use them in production lines (Statheropoulos et al. 2005; Strangl et al. 2018). Research on this subject leads to the conclusion that this bad smell is not caused by a single substance but is caused by a complex mixture of substances (Cabanes and Fullana 2021). The odour of plastics can come from several sources from the virgin starting material that can react or degrade during polymerisation generating volatile compounds with undesirable odours, additives that can volatilise at low temperatures causing odours, during processing of the materials volatile compounds can be produced that generate odours, during the use of plastics new odours may be generated, during the packaging of foodstuffs undesirable odours may occur due to the aggressive treatments necessary to preserve foodstuffs, or because these lead to chemical reactions that release compounds that generate unpleasant odours. In addition to the above, there is the odour of recycled plastics.

In the case of recycled plastics, the odour may come from compounds that may have been generated by environmental or thermal degradation after use, but also those produced during the recycling process such as the degradation of the polymer itself, plastic additives or substances with which the material has been in contact. In addition, because polymers can absorb volatile compounds from their previous use, substances such as fragrances may be present.

Other less common causes may be improper storage of the containers (humidity, temperature) and presence of other containers emitting substances, which could cause undesirable odours.

In the literature, we can find articles dealing with different washing systems consisting of water and a detergent to try to eliminate bad odour; however, as the number of compounds is so high, it is very difficult to treat all polymers with the same system, as the type of plastic conditions the washing system and the detergent to be used (Boz Noyan et al. 2022; Roosen et al. 2022).

High-density polyethylene, HDPE, is one of the most widely used polymers in the packaging industry (Fuller et al. 2020). The aim of the work is to develop an efficient process to eliminate or significantly reduce odours associated with high-density polyethylene (HDPE) from waste management plants, in order to enable its reuse in industrial processes and promote a circular and sustainable economy for plastics.

## Materials and methods

### Source material

**Fig. 1:**
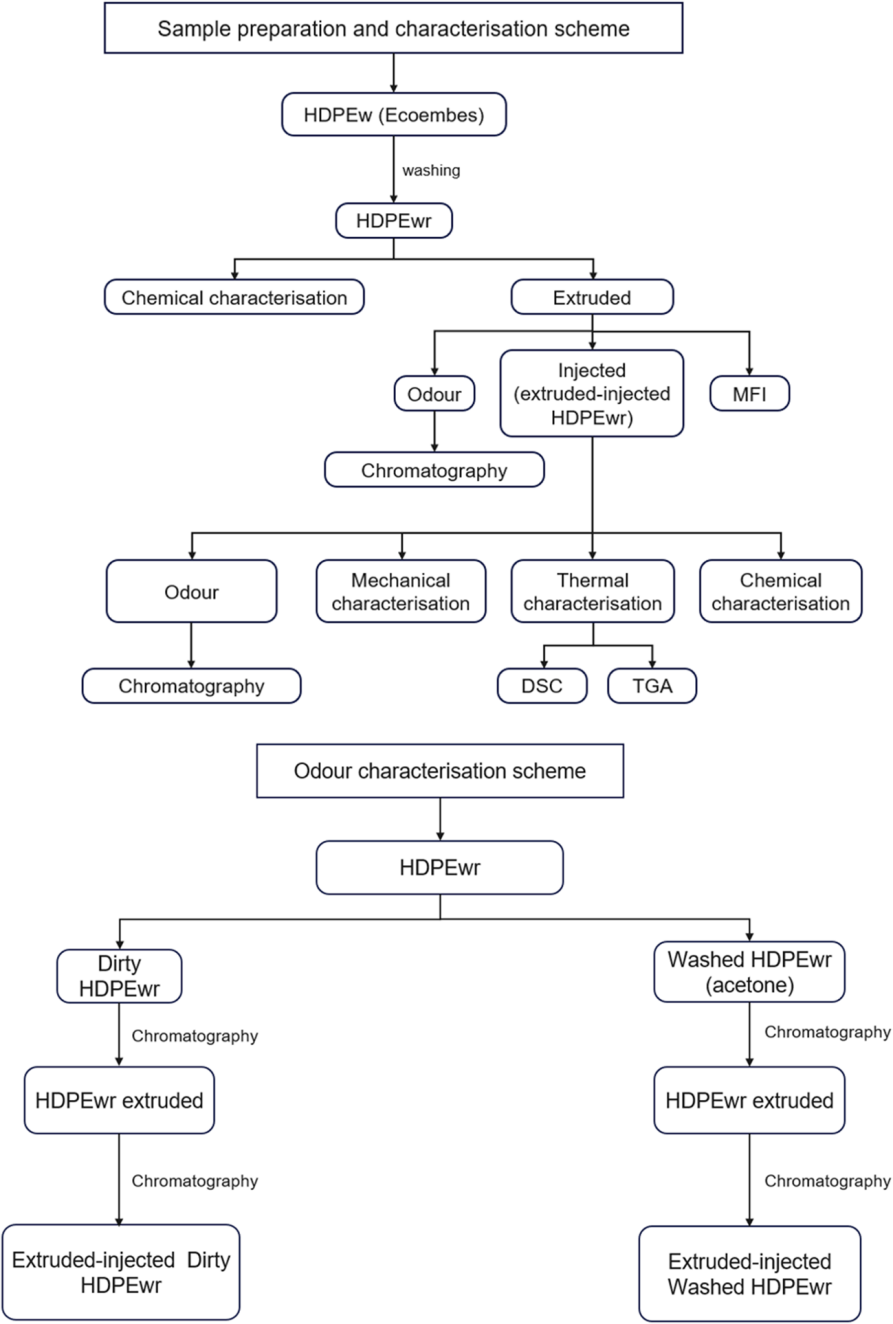
Overall scheme of the process

The company Ecoembes (Spain), dedicated to the management, treatment and recycling of waste, has implemented a solid urban waste (SUW) collection system using four different coloured containers to identify each of the materials to be collected. The high-density polyethylene waste comes from mixed post-consumer packaging waste collected in the yellow containers. Thus, plastic packaging, cans and cartons go into the yellow container. Once there, plastic packaging, metal packaging and briks are separated at packaging sorting plants, where light packaging is separated into at least three fractions: metals (steel and aluminium), plastics (PET, HDPE, film, mixed waste) and briks. By the method of the difference in density of the polymers, a mixture of HDPE, LDPE, PP, PS and PVC is separated. The small difference in the density value of these compounds is sufficient to separate them. Finally, the HDPE source material was cut into flakes of approximately 1 × 1 cm^2^ (Fig. 1).

**Fig. 2:**
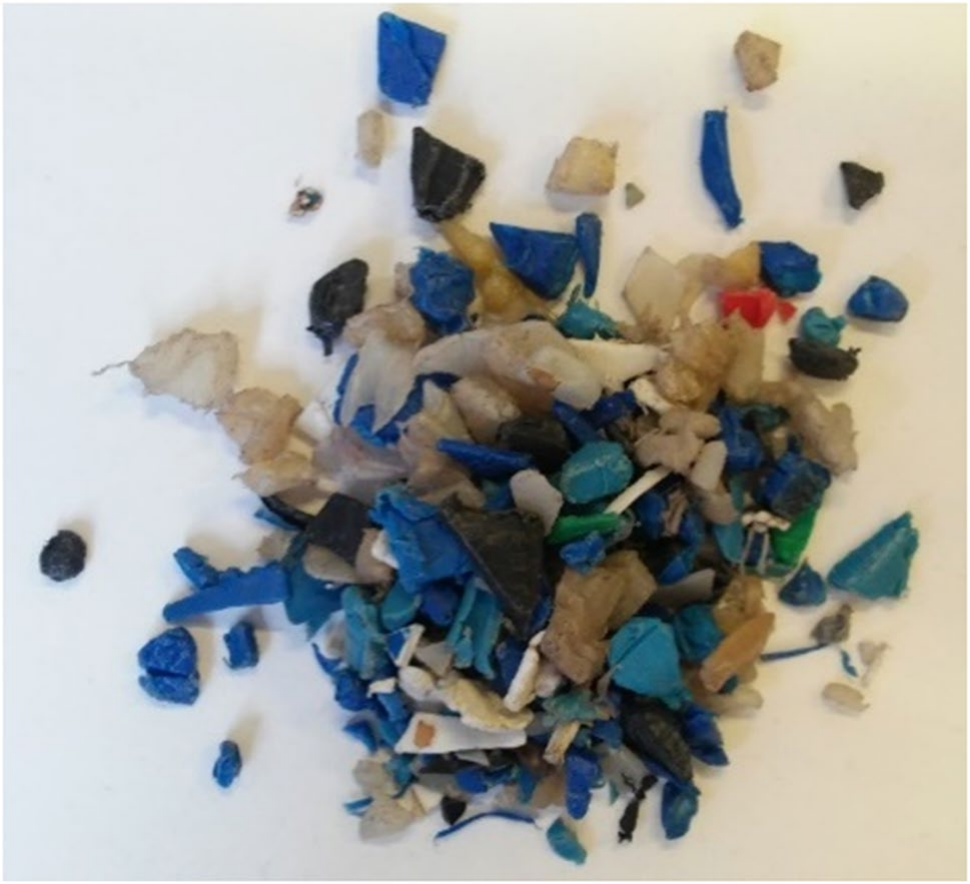
HDPEw sample with traces of other materials supplied by Ecoembes

The source material supplied by the company Ecoembes (Spain) contained high-density polyethylene (HDPEw) with small amounts of other materials (Fig. 2).

#### Preparation of starting material

The HDPEw starting material together with other materials was washed with chloroform (to remove resin, adhesives, grease, etc.) and magnetic stirring for 5 min. Subsequently, it was placed in a soap and water solution with magnetic stirring for a further 5 min and finally rinsed with distilled water to remove dirt.

After cleaning, the sample was dried in an oven at 40 °C for 10 h (HDPEwr sample). The drying temperature of 40 °C is due to the fact that HDPE undergoes oxidation at 50 °C, but can even degrade at ordinary temperatures in the presence of light. For this reason, antioxidants are usually incorporated.

### Processing of starting material

For the characterisation of the HDPEwr starting material, it was extruded in a twin-screw extruder L/D 24 from Dupra S.L. (Alicante, Spain) with a temperature profile of 185–190–195 °C (extruded HDPEwr sample) and then injected in the form of standardised test tubes in a Sprinter 11t injector from Erinca S.L. (Barcelona, Spain), using a temperature profile of 185–190–195 °C (extruded-injected HDPEwr sample).

#### Melt flow index (MFI)

The MFI is a measure of the flow rate of the plastic material and is inversely proportional to the viscosity and molecular weight of the polymer. The MFI is determined as the amount by weight of polymer extruded through the nozzle in a time *t* (usually 10 min). It is measured in g/10 min.

The extruded material (extruded HDPEwr) was tested for melt flow index (MFI) with a plastometer from AtsFarr S.p.A. (Vignate, Italy) under the following conditions: at 190 °C and a weight of 10 kg, at 210 °C and a weight of 10 kg, at 220 °C and a weight of 10 kg, at 230 °C and a weight of 10 kg, at 240 °C and a weight of 10 kg.

### Characterisation of samples

#### Chemical characterisation

The HDPEwr sample and the extruded-injected HDPEwr material were characterised by Fourier transform infrared spectroscopy using a PerkinElmer Spectrum BX spectrometer (PerkinElmer España S.L., Madrid, Spain).

Infrared spectroscopy tests were performed by 20 scans between 600 and 4000 cm^−1^ with a resolution of 16 cm^−1^ using an attenuated total reflectance (ATR) accessory, indicated for samples with poor transparency. The spectra obtained by FTIR allow monitoring of polymerisation reactions, characterisation of polymer structures, and determination of polymer functional groups or degradation processes.

#### Thermal characterisation

The extruded-injected HDPEwr material was characterised by differential scanning calorimetry (DSC) with a Mettler Toledo 821 (Mettler Toledo, Schwerzenbach, Switzerland) and thermogravimetric analysis using a Linseis TGA PT1000 (Selb, Germany).

##### Differential scanning calorimetry (DSC)

Samples of 5–10 mg were used to carry out the DSC tests. The first test was performed in nitrogen atmosphere, using a heating and cooling rate of 10 °C. First, heating from 30 to 170 °C was performed, followed by cooling to 80 °C and then heating to 270 °C; this DSC test allowed to remove the thermal history of the sample and to obtain information about the melting of the material. The second test was carried out in air atmosphere, first with a heating rate of 10 °C/min from 30 to 190 °C, followed by an isotherm at 190 °C for 30 min using air atmosphere; this test allowed to obtain the onset of degradation by determining the oxidation induction time (OIT).

##### Thermogravimetric analysis (TGA)

Thermogravimetric analysis (C/min) is a method of thermal analysis in which the mass of a sample is measured over time as the temperature changes or at a constant temperature for a given time. It is used to determine the thermal stability of a material, composition, purity, decomposition reactions, decomposition temperature and absorbed moisture content of products.

The thermal stability of the samples was evaluated by TGA; the tests were carried out with a sample weight between 15 and 20 mg in alumina crucibles of 70 ηL capacity. The samples were heated from 35 to 700 °C with a heating rate of 10 °C/min and in nitrogen atmosphere (30 mL/min). The onset of degradation temperature was taken at 5% degradation (T5%), and the maximum decomposition rate (TMAX) was also determined at the minimum peak of the first derivative of the TGA curves (DTG).

#### Mechanical characterisation

The mechanical characterisation of the extruded-injected HDPEwr specimen was carried out by Charpy tensile and impact tests. For the tensile tests, an Ibertest universal testing machine model ELIB 30 (Madrid, Spain) with a load cell of 5 kN at a speed of 10 mm/min was used and five specimens of each sample were tested, as indicated in the UNE-EN ISO 527 standard.

The Charpy impact test was performed with a Charpy Pendulum from Metrotec S.A. (San Sebastian, Spain), using a 1 J pendulum and the specimens were notched with type A geometry according to UNE-EN ISO 179–1:2011.

### Odour testing

#### Gas chromatography

Odour problems in plastics are generally caused by substances with high volatility, i.e., molecules that vaporise at relatively low temperatures (< 100 °C). Some of these substances can be found in concentrations below mg/kg, making it necessary to use chromatographic techniques with universal detection systems such as mass spectrometry (MS). Gas chromatography-mass spectrometry (GC–MS) is a technique that combines the separation capacity of gas chromatography with the sensitivity and selectivity of the mass detector to quantify volatile and semivolatile organic compounds in samples with a high degree of effectiveness.

The HDPEw sample has an odour, which makes it a material whose final applications are limited. For this reason, a gas-mass chromatography (GC–MS) analysis was carried out to detect and quantify the volatile compounds causing this possible odour in the samples.

The GC–MS equipment used was an Agilent 5977A mass spectrometer (Agilent Technologies Spain, Las Rozas de Madrid, Spain) with low-resolution quadrupole analyser, with a gas chromatograph (Agilent 7890B) for capillary columns (spli/splitless, pulsed split and pulsed splitless) and GC–MS interface. The initial flow rate was 1 mL/min, with a vial shaking speed of 500 rpm, an incubation temperature of 80 °C, and in constant flow mode. The interface temperature was 280 °C. The injection mode was splitless 60 mL/min for 1 min. The thermal cycle was an initial temperature of 40 °C for 5 min, followed by a rate of 6 °C/min until 200 °C was reached, and then, the heating rate was increased to 20 °C/min until 300 °C and maintained at that temperature for 4 min. The results obtained were analysed with the Agilent “Qualitative” program and the “Unknowns Analysis” deconvolution program.

#### Odour testing by means of olfactory panels

This technique consists of selecting a group of people (olfactory panel) to sniff the odour sample. The analysis of the samples was carried out by adapting the VDA 270 standard, used for odour assessment in the automotive industry. For this purpose, 10 g of each sample was placed in a 1-L glass container and kept closed at 80 °C for 2 h. After cooling to 60 °C, ten volunteers (three males and seven females) aged 21–56 years rated the overall odour of the samples on a scale of 1—not perceptible, 2—perceptible, not annoying, 3—clearly perceptible, not annoying, 4—annoying, 5—very annoying, and 6—intolerable. The mean value of these ratings was calculated and the degree rounded down was specified. All volunteers had no known illness at the time of sensory evaluation (Prado et al. 2020).

## Results and discussion

### Sample preparation

The HDPEw sample, supplied by Ecoembes, was washed and dried (HDPEwr sample) and fed into a twin-screw extruder L/D 24 of Dupra S.L. (Alicante, Spain), and depending on the MFI (melt flow index), the resulting material was added to a Sprinter 11t injector of Erinca S.L. (Barcelona, Spain) resulting in the extruded-injected HDPEwr sample.

#### Nomenclature of samples

The samples under study, their nomenclature and origin, are listed in Table 1.

**Table 1:** Nomenclature and origin of samples

Nomenclature	Provenance and treatment
HDPEw	Waste supplied by Ecoembes
HDPEwr	Waste washed with chloroform, soap and water
Extruded HDPEwr	Washed and extruded waste
Extruded-injected HDPEwr	Washed and extruded-injected waste

### Characterisation of samples

#### Characterisation of the starting material HDPEwr

Controlling the selection of recycled materials is a very important process on which the final quality of the product depends. Poor quality can be attributed to the presence of grease, detergents, printing inks, mixing with other polymers such as adhesives, or even the reprocessing of virgin material (Brent 2006). One solution to this problem is to study the materials present in the HDPEwr sample. For this purpose, it is proposed to characterise the starting material chemically to detect the presence of other polymeric materials that may influence its properties.

##### Chemical characterisation of HDPEwr

Chemical characterisation was carried out using FTIR spectra. These spectra allow us to determine the functional groups of the polymers or the degradation processes they have undergone. For this purpose, 12 fragments of the HDPEwr sample were chemically characterised to determine the presence of HDPE and other materials. The following figures show the compounds present in the selected sample and the FT-IR spectrum of HDPEwr together with the other materials detected in the sample supplied by Ecoembes.

**Fig. 3:**
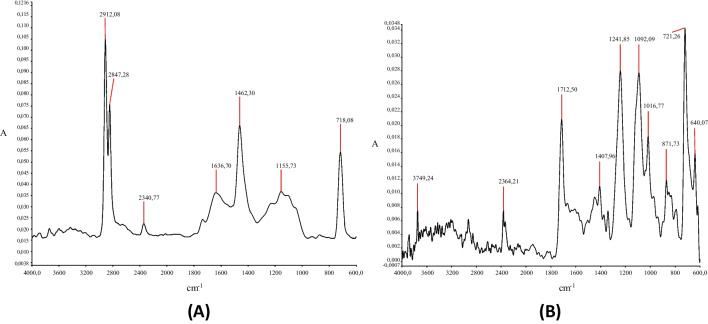
FT-IR spectra. **A** High-density polyethylene (HDPE) and **B** polyethylene terephthalate (PET)

The spectra obtained show the different chemical compositions of the polymers present (Fig. 3).

The HDPE spectrum in Fig. 3 and the bands assigned to that spectrum (Table 2) show that this is a contaminated HDPE material. In the HDPE spectrum (Fig. 3), clusters of bands corresponding to symmetric and antisymmetric stretching of the -CH_2_ bonds around 2912 and 2847 cm^−1^, C–C bond at 1155 cm^−1^ and -CH_2_ torsional vibrations at 718 cm^−1^ are clearly observed (Fernanda and Batallas 2016; Gulmine et al. 2002; Ricardo and Cabra 2017).

**Table 2:** Band assignments in the FT-IR spectrum (high-density polyethylene (HDPE))

Bands (cm^−1^)	Assignment
2912	Symmetrical stretching (-CH_2_-)
2847	Asymmetric stretching (-CH_2_-)
2340	Presence of contaminant or interference
1630	Impurities or any unsaturation
1462	Group bending (-CH_3_)
1155	Link C–C
718	Torsional vibration (-CH_2_-) associated with crystalline structures in the polymer

Table 3 also reports bands describing the crystalline and amorphous properties of PET, as well as the presence of water and contaminants. The spectrum of PET in Fig. 3 and the bands assigned to that spectrum (Table 3) show an angular deformation -CH_2_ at 1407 cm^−1^ and a C–C bond vibration at 1016 cm^−1^ (Atkinson et al. 2000; Cobbs and Burton 1953; Liang and Krimm 1959; Sammon et al. 2000).

**Table 3:** Band assignments in the FT-IR spectrum (polyethylene terephthalate (PET))

Bands (cm^−1^)	Assignment
3749	Presence of water
2364	Presence of contaminants
1712	Stretching (C = O)
1407	Angular deformation (-CH_2_)
1241	Bending vibration (C = O)
1092	Stretching link C–O–C
1016	Link vibration (C–C)
871	Aromatic ring bending vibration in terephthalate
721	Torsional vibration (C–C)

In addition, Tables 2 and 3 also describe the 1712 cm^−1^ band ratio, generally applied to describe the oxidative process in recycled plastics (Schmidt 1963; Stangenberg et al. 2004).

**Fig. 4:**
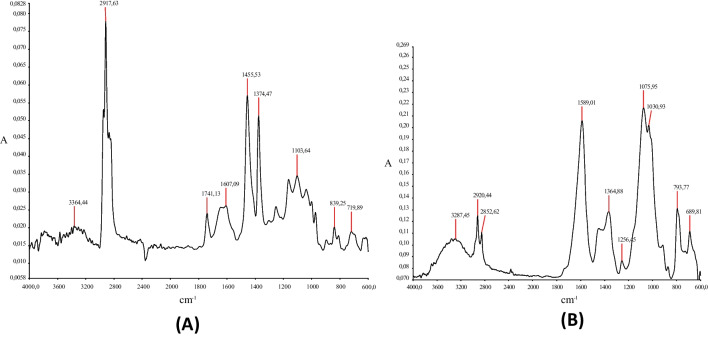
FT-IR spectrum. **A** Elongated white fragment, polypropylene (PP), and **B** brown fragment, flexible polyurethane foam

The PP spectrum (Fig. 4) and the bands assigned to that spectrum (Table 4) show the possible presence of additives and contaminants. Likewise, the PP spectrum (Fig. 4) shows a band corresponding to asymmetric stretching of the -CH_2_ bonds at 2917 cm^−1^ and tensile stress vibrations of the C–C bond between 1108 and 719 cm^−1^ (Dorai and Kushner 2003; Ricardo and Cabra 2017; Sciarratta et al. 2003; Socrates 2001).

**Table 4:** FTIR band assignments in the spectrum (polypropylene (PP))

Bands (cm^−1^)	Assignment
3354	Presence of humidity
2917	Asymmetric stretching (-CH_2_-)
1741	Probable presence of additives containing (C = O)
1607	Bending vibrations (C = C) in aromatic impurities or unsaturations present
1455	Group bending vibrations (-CH_2_-)
1374	C-H bond bending vibrations
1108, 839, 719	(C–C)-bond bending vibrations

The FTIR spectrum obtained for flexible polyurethane (PU) foam (Fig. 4) shows the different PU bands that can be identified using literature data (Ricardo and Cabra 2017). These bands are listed in Table 5; thus, the corresponding band (N–H) is observed between 3500 and 3300 cm^−1^, the bands at 2920 and 2850 cm^−1^ correspond to symmetric and asymmetric stretching (-CH_2_) and the band at 793 cm^−1^ to bond vibrations (C-N) (Socrates 2001).

**Table 5:** FTIR band assignments in the spectrum (flexible polyurethane foam)

Bands (cm^−1^)	Assignment
3287	N–H
2920 and 2852	Symmetric and asymmetric stretching (-CH_2_-)
1509	Deformation of the C = C bond if aromatic units are present
1364	Bending vibrations (-CH_2_) and could also be presence of urethane groups
1256	Bond stretching (C-O)
1075 and 1080	C–O–C bond stretching
793	Bond vibration (C-N)
689	Vibration and C–C bending

After FTIR analysis of the sample, it was possible to identify the material (Table 6). Most of the fragments analysed were HDPE, although other materials such as polypropylene (PP), polyethylene terephthalate (PET) and flexible polyurethane foam were detected in small quantities. These results indicate that the HDPEw material is mainly a mixture of polyethylene fragments, but with the presence of other plastics.

**Table 6:** Material identification by FTIR of selected fragments (HDPEwr sample)

Sample	Fragment	Material
A	Green	Polyethylene
	Triangular white	Polyethylene
	Grey	Polyethylene terephthalate
	Elongated white	Polypropylene
	Brown	Flexible polyurethane foam
	Blue	Polyethylene
B	Dark green	Polyethylene
	Red	Polypropylene
	Blue	Polyethylene
	White	Polyethylene
	Light green	Polyethylene
	Black	Polyethylene

#### Extruded HDPEwr material characterisation

Once the HDPEwr material had been analysed, it was extruded and its melt flow index (MFI) was determined in order to evaluate a subsequent injection of the material.

##### Extruded HDPEwr sample extruded HDPEwr flow index

The (MFI) allows us to determine the flow rate of extruded HDPEwr material. The polymers with a low melt flow index show higher cohesive strength and elasticity, but are more difficult to process due to their high viscosity. A low average molecular weight equates to high melt flow rates, with the melt having a low viscosity, ideal for injection moulding processes.

The extruded HDPEwr sample was tested under different test conditions, varying the temperature. The values obtained are shown in Table 7. In this table, it can be seen that as the temperature increases, the flow index increases, since this is a material whose viscosity is highly influenced by the temperature. Furthermore, it can also be seen that the values are between 1.0 and 3.6 g/10 min, which indicates that the selected residues are from parts that have mainly been obtained by extrusion or extrusion-blowing. If we compare the MFI values obtained with other thermoplastics reported in the literature, we find lower values than those obtained in this work (Hamad et al. 2012; Völtz et al. 2023), although the measurement temperatures are higher, which explains these high values (Dewi et al. 2023), indicating the greater or lesser ease of flow in extrusion-injection. Likewise, as MFI is inversely proportional to viscosity, it is observed that as the temperature increases, MFI increases and the viscosity of the material fluid decreases, facilitating the injection of the material.

**Table 7:** Melt flow index (MFI) values of extruded HDPEwr material at different temperatures

Temperature (°C)	Weight (kg)	MFI (g/10 min)
190	10	1.05
210	10	2.17
220	10	2.87
230	10	3.03
240	10	3.63

Once the extruded HDPEwr sample flow rate was determined, the extruded-injected HDPEwr sample was injected and characterised.

#### Chemical characterisation of the extruded-injected HDPEwr sample

**Fig. 5:**
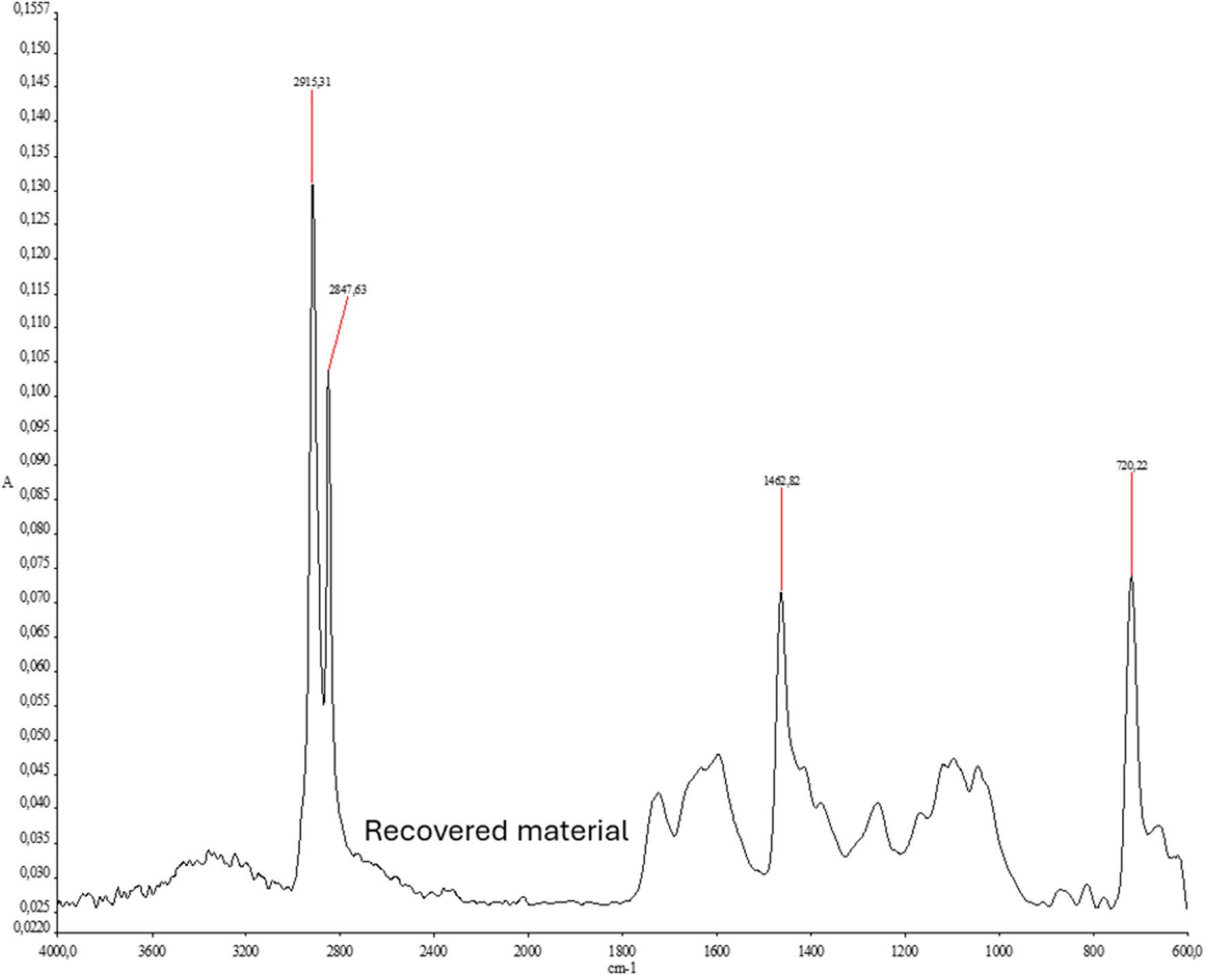
FT-IR spectrum: extruded-injected HDPEwr sample

The extruded-injected HDPEwr material was analysed by FTIR. The infrared spectrum is shown in Fig. 5; it can be seen that it is a typical HDPE spectrum; however, low-intensity bands are observed at 1750, 1600, 1250, 1070 and 1020, which may be due to small amounts of dirt residues present in the starting sample and/or the minority presence of other plastics, as seen in the previous peaks.

The HDPE spectrum (Fig. 5) and the bands assigned to that spectrum (Table 8) show that the HDPEwr material is less contaminated than the samples analysed previously. In the HDPEwr spectrum (Fig. 5), groups of bands are clearly observed, which are collected and assigned in Table 8 and correspond to symmetrical and asymmetrical stretching (-CH_2_-) at 2915 and 2847 cm^−1^, bending band of the (-CH_3_) group and bending vibrations of the C–C bond.

**Table 8:** FTIR band assignments in the extruded-injected HDPEwr spectrum

Bands (cm^−1^)	Assignment
2915	Symmetric stretching (-CH_2_-)
2847	Asymmetric stretching (-CH_2_-)
1462	Group bending (-CH_3_)
720	Bending vibrations of the C–C bond

#### Thermal characterisation of extruded-injected HDPEwr sample

##### Differential scanning calorimetry of the extruded-injected HDPEwr sample

Semicrystalline polymers, such as polyethylene (PE), polypropylene (PP), and polyethylene terephthalate (PET), are systems containing a crystalline phase and an amorphous phase. Differential scanning calorimetry (DSC) is used to characterise these semicrystalline materials. Differential scanning calorimetry is one of the thermal analysis techniques that allows among other things qualitative and quantitative identification of materials, degradation studies, and study of previous thermal processes accumulated by the material (“Thermal Analysis of Pharmaceuticals” 2006).

**Fig. 6:**
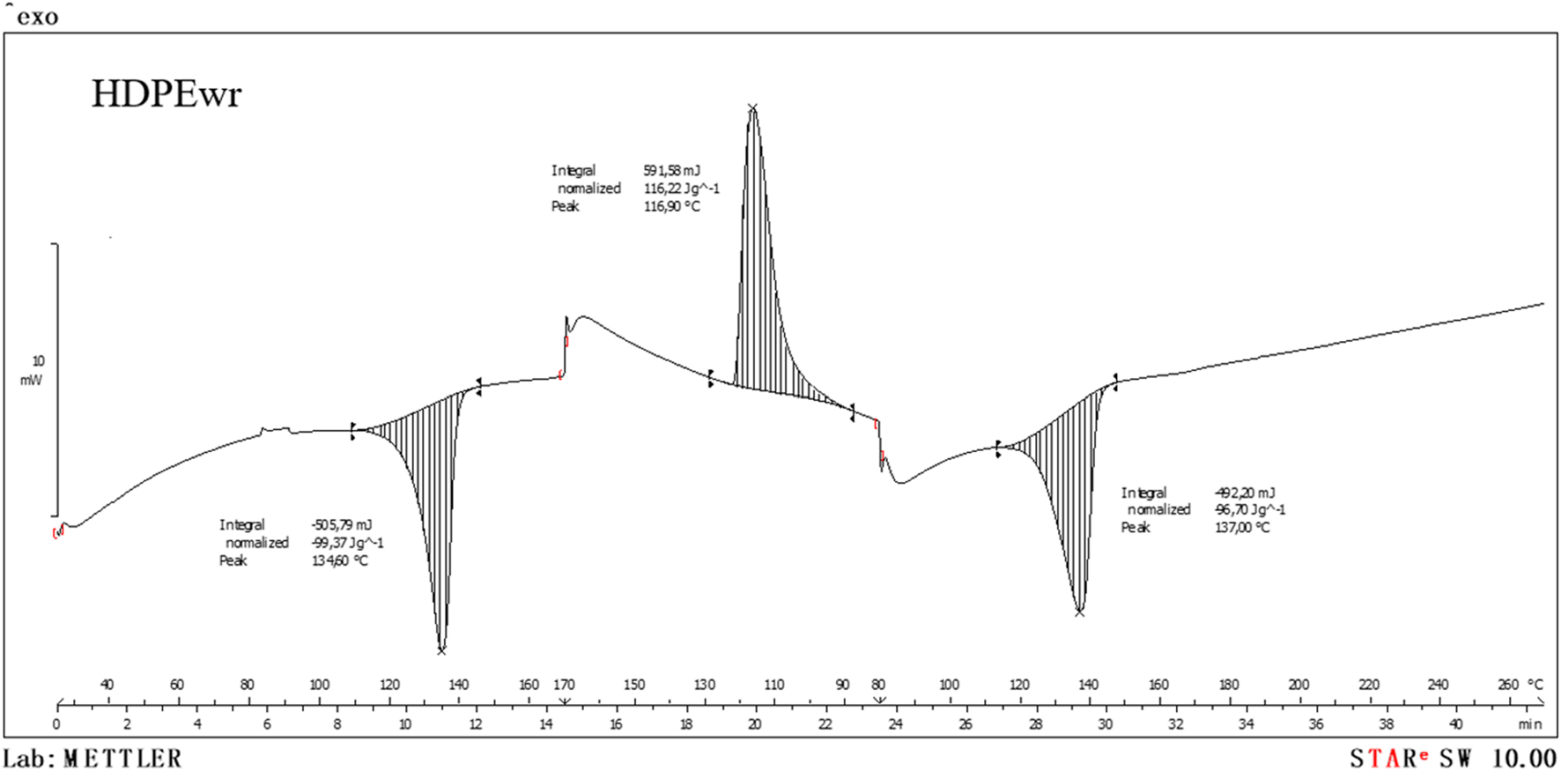
Dynamic and N_2_ atmosphere calorimetric graph of extruded-injected HDPEwr material

In view of Fig. 6, corresponding to the first DSC test in nitrogen atmosphere, the extruded-injected HDPEwr sample presents a wide temperature gradient, the maximum temperature giving the actual melting temperature. This is the most frequently used melting temperature for semicrystalline polymers; in our case, it is determined from the second heating in which it can be seen that the melting peak is at 137.0 °C, typical melting temperature of a high-density polyethylene (Moreno et al. 2017); in addition, no other melting peak is observed, which indicates that the sample does not present a large amount of other semicrystalline plastic materials as previously discussed.

Another important characteristic of melting in semicrystalline polymers when using DSC is the enthalpy of fusion, Δ*H*_f_. The value of Δ*H*_f_, determined by DSC, indicates only the amount of crystallinity present in the sample and not the inherent value of the enthalpy of fusion of a fully crystalline polymer, Δ*H*_f100_. If the enthalpy of fusion of the fully crystalline polymer is known (Δ*H*_f100_ of HDPE 293 J/g), the degree of crystallinity of an extruded-injected HDPEwr polymer sample can be determined as follows:

1 $${X}_{C}(\% \text{crystallinity})=\frac{\Delta {H}_{\text{f}}}{w\Delta {H}_{\text{f}100}}\cdot 100$$

In view of the calorimetric curves (Fig. 6), an enthalpy of fusion of 96.70 J/g is obtained and therefore a crystallinity of 33.3%, which was calculated with Eq. 1, indicates that approximately 33.3% of the polymeric material is in crystalline form.

2 $${X}_{C}(\% \text{crystallinity})=\frac{\Delta {H}_{\text{f}}}{w\Delta {H}_{\text{f}100}}\cdot 100=\frac{\text{96,70}}{293}\cdot 100=33.3\%$$

Crystallinity is related to the mechanical strength of the thermoplastic. Semicrystalline thermoplastics have high mechanical strength, are stiffer, and are more resistant to creep and heat than amorphous thermoplastics. This is because the crystalline regions provide a more ordered and rigid structure to the material, which gives it greater rigidity and resistance to deformation. On the other hand, they are less impact-resistant and less flexible. This is due to the presence of crystalline regions, within the amorphous matrix, which can act as initiation points for fracture. In addition, the presence of amorphous regions in the material can provide some flexibility and impact resistance.

**Fig. 7:**
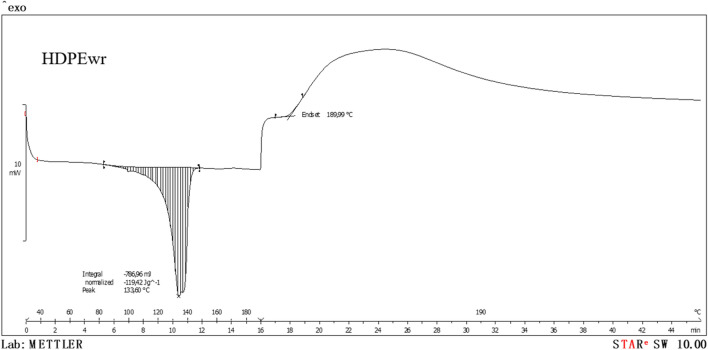
Dynamic-isothermal and air-atmosphere calorimetric graph of extruded-injected HDPEwr material

From the second air atmosphere DSC test (Fig. 7), the oxidative induction time (OIT) of the extruded-injected HDPEwr material is determined.

The oxidative induction time (isothermal OIT) is a relative measure of the resistance of a (stabilised) material to oxidation, determined by calorimetric measurement of the time interval until the onset of oxidation of the material. The sample is heated in a dynamic air atmosphere at a constant rate until the oxidation temperature is revealed by an exothermic deviation in the DSC heat flow curve. The dynamic OIT is the temperature at which the oxidation reaction starts. Due to the dynamic temperature control, the onset of oxidation is often indicated by a sharp increase in the DSC heat flux curve in the exothermic direction during the DSC measurement.

In view of Fig. 7 corresponding to the DSC in air atmosphere of the extruded-injected HDPEwr sample, it can be seen that the OIT occurs in a time of 2 min and 10 s and at a temperature of 190 °C, fundamental data to evaluate the thermal stability and degradation of the polymer in real conditions of use. The degradation temperature 190 °C of extruded-injected HDPEwr is the maximum temperature at which extruded-injected HDPE can be processed without losing its physical and chemical properties. Degradation reduces the molecular weight of the polymer because it breaks the covalent bonds of the chains formed during polymerisation.

In this case, the HDPEwr sample appears to be susceptible to oxidation at the relatively moderate temperature of 190 °C, which may have important implications for its application and processing in terms of temperature selection and the need for oxidation protection measures, such as the use of antioxidants or modifications in the manufacturing process.

##### Thermogravimetric analysis extruded-injected HDPEwr

Thermogravimetric analysis (TGA) is a thermal analysis method to determine the thermal stability of a material, the decomposition temperature and mass loss among other parameters.

**Fig. 8:**
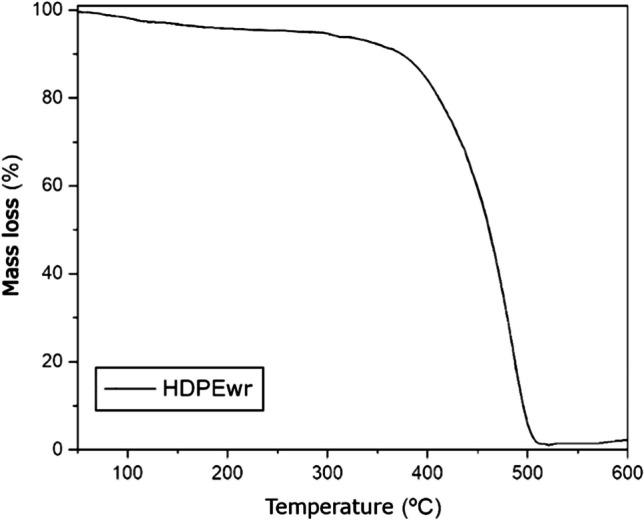
TGA curves of extruded and injected HDPEwr samples

Figure 8 shows the degradation profile that HDPEwr undergoes. The degradation of HDPEwr ends up being almost 100%, with almost all of it being converted into volatile products. Figure 8 shows that as the temperature increases, there is a weight loss in the thermogram, indicating the decomposition of the material. This weight loss increases as the temperature approaches *T*_max_ and reaches its maximum around *T*_f_.

Further thermogravimetric analysis of the extruded-injected HDPEwr sample yields the main TG curve data such as the start and end temperature of degradation, the temperature of maximum decomposition rate, and the final mass of the sample which are given in Table 9.

**Table 9:** Melting temperature (*T*_m_), crystallisation temperature (*T*_c_), enthalpy of fusion (Δ*H*_m_), crystallinity (*X*_c_), onset of degradation temperature (*T*_5%_), maximum decomposition rate temperature (*T*_max_), final decomposition temperature (*T*_f_) and mass % at the end of decomposition (*M*_f_) of the sample

	DSC				TGA			
Material	*T*_m_ (°C)	*T*_c_ (°C)	Δ*H*_m_ (J/g)	*X*_c_ (%)	*T*_5%_ (°C)	*T*_max_ (°C)	*T*_f_ (°C)	*M*_f_ (%)
Extruded-injected HDPEwr	135.6	114.4	166.5	56.8	282	485	506.0	1.1

In view of the thermogram, the temperature at which 5% of the total has been lost (*T*_5%_) has been determined, which is 282 °C and *T*_f_ which indicates the end of the process and the temperature at which the maximum weight loss occurs, 506 °C; this means that at this temperature, most of the material has decomposed into volatile products. *T*_max_ is the temperature at which the maximum rate of decomposition occurs, 485 °C. This is the temperature at which the decomposition rate of the material is maximum.

According to the literature, in the complete absence of oxygen, polyethylene is stable up to 290 °C. Between 290 and 350 °C, it decomposes and gives lower molecular weight polymers, which are usually thermoplastics or waxes, but little ethylene is produced. At temperatures above 350 °C, gaseous products are produced in increasing amounts, the main product being butylene (Peacock 2000).

#### Mechanical characterisation extruded-injected HDPEwr

The mechanical characterisation was carried out by means of tensile and Charpy impact tests. The tensile test consists of applying an axial stress to a specimen until it breaks. These tests allow us to determine the strength and deformation properties under tensile stress. The Charpy test consists of a hammer striking the centre of the specimen to break it in half. This test allows us to determine the toughness of a material, or the tendency to resist breakage, when subjected to an impact.

The Charpy tensile and impact test values for extruded-injected HDPEwr material are given in Table 10.

**Table 10:** Tensile test values of HDPEwr material injected by extrusion

Test tube	Resistance (MPa)	Lengthening (%)	Absorbed energy-Charpy (kJ/m^2^)
1	19.0	149	36.5
2	21.7	131	30.2
3	19.1	155	29.6
4	21.4	115	32.1
5	22.6	97.5	40.1
Mean	21.2	125	33.7
Standard deviation	1.49	24.4	4.49

The tensile test indicates that the extruded-injected HDPEwr sample has an average strength of 21.2 MPa and an elongation of 125%. These values are similar to other recycled HDPE **(***Tipos de Plásticos HDPE | Catálogo | ACTECO* n.d.). On the other hand, the Charpy test allows us to determine the energy absorbed by the specimen through the Charpy impact; in our case, the average impact energy absorbed by the extruded-injected HDPEwr specimen is 33.7 kJ/m^2^.

### Odour analysis of samples

#### Gas chromatography

The odour of plastics can come from a variety of sources from the virgin starting material to additives or even odours that may be generated during the use of the plastic. Many researchers have conducted research aimed at eliminating or reducing the odour of plastics (Chen et al. 2020; Karaagac et al. 2021; Strangl et al. 2019).

In the case of recycled plastics, the odour may come from products generated by environmental or thermal degradation, from volatile compounds impregnated in their previous use, from inadequate storage of the packaging, etc.

During the preparation of the samples, it was observed that the samples, once extruded and subsequently injected, lose part of this odour. For this reason, it was decided to analyse the samples by GC–MS, to check if any of the volatile compounds were responsible for the odour and even to analyse the influence of washing the sample with acetone to assess the intensity of the odour with respect to the other samples.

In order to detect the fraction of volatiles during the different processes, a gas-mass chromatography (GC–MS) analysis of the samples was carried out. On the one hand, the influence of the extrusion and injection processes on odour was analysed: samples HDPEwr, extruded HDPEwr, and extruded and injected HDPEwr. On the other hand, the influence of washing with acetone for 15 min and magnetic agitation was analysed to favour the elimination of volatiles by dragging both acetone and other volatiles existing in the samples. This treatment results in three samples: HDPEwr (acetone), HDPEwr extruded (acetone), and HDPEwr extruded-injected (acetone). It is important to leave the acetone-treated samples in the fume hood until complete removal of acetone volatiles, due to its flammability. The above six samples were subjected to gas chromatography-mass spectrometry (GC–MS) to detect and quantify the volatile compounds and their possible relationship with the odour.

**Fig. 9:**
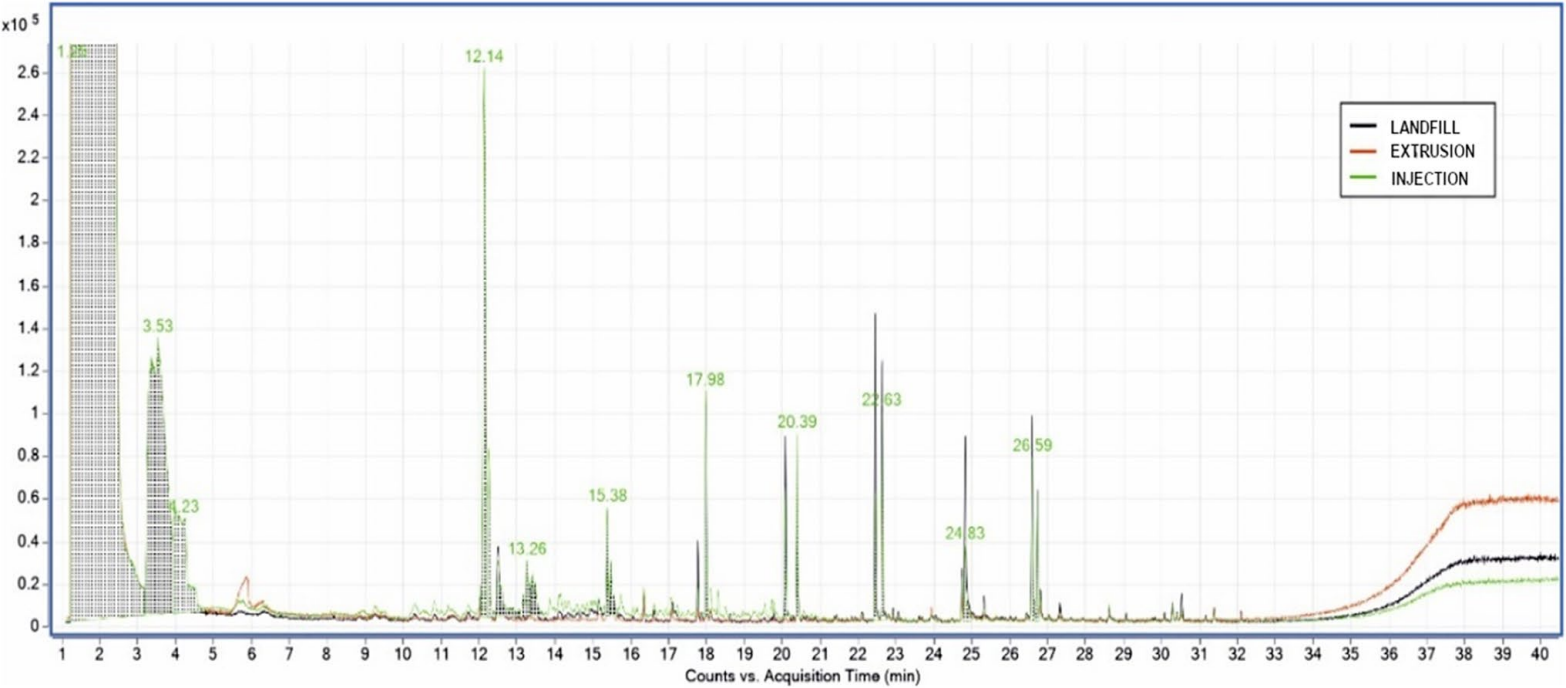
Chromatogram of sample materials, extruded and injected

The samples analysed present the following fraction of volatiles: dodecane, 1-tetradecane, phenol2-4 bis and hexadecene as shown in Table 11 and Fig. 9. However, when trying to relate the chromatographic results with the sensory perceptions when smelling the different samples, the data obtained do not allow establishing a relationship with odour. There are many volatile compounds in each sample, but not all of them have an odour.

**Table 11:** Comparison of the most significant retention peaks of dirty and clean samples, normalised values at 20.39 min

Retention time (min)	HDPEwr			HDPEwr clean with acetone			Composite identification	Formula
	HDPEwr Landfill	HDPEwr Extruded	HDPEwr Injected	HDPEwr Landfill	HDPEwr Extruded	HDPEwr Injected		
17.98	205	102	122	40	80	0	Dodecane	C_12_H_26_
22.45	295	135	70	72	60	22	1-Tetradecene	C_14_H_28_
24.83	175	20	41	16	0	0	Phenol 2–4 bis	C_14_H_22_O
26.59	198	140	88	78	80	36	Hexadecene	C_16_H_32_

Table 11 and the figures above show that the intensity of the peaks corresponding to the starting and subsequently washed HDPEwr, extruded HDPEwr, extruded-injected HDPEwr and extruded-injected HDPEwr samples decreases the intensity of the peaks corresponding to the different compounds. This phenomenon is due in principle to the entrainment of certain compounds by chloroform, water and soap in the starting sample. Likewise, the heat treatments to which the sample is subjected during the extrusion and injection processes favour the elimination of volatiles with increasing temperature.

With the results obtained, the most representative peaks were normalised, taking the band 20.39 as the reference value.

**Fig. 10:**
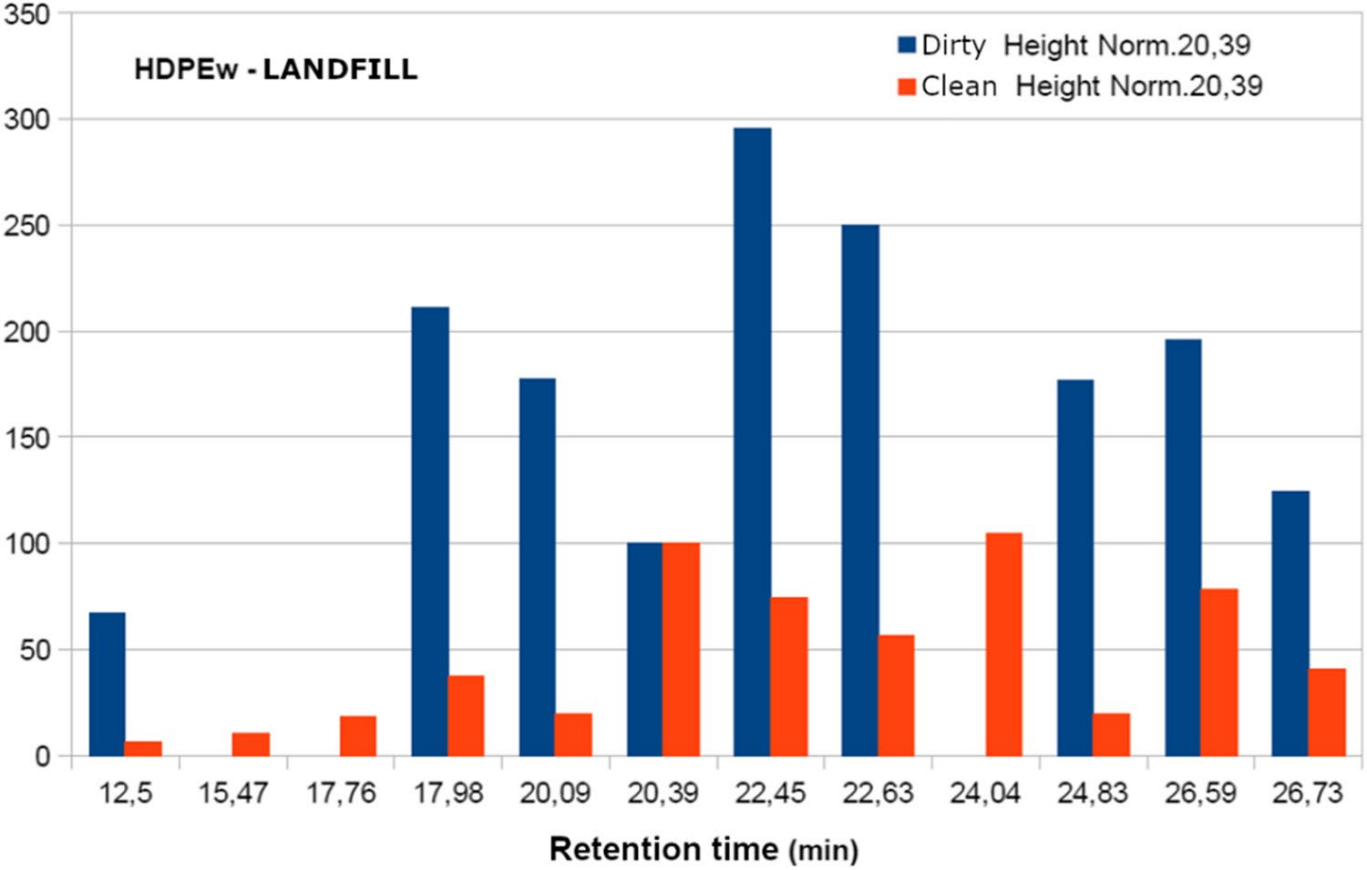
Normalisation of the peaks of the HDPEw sample from the value 20.39

**Fig. 11:**
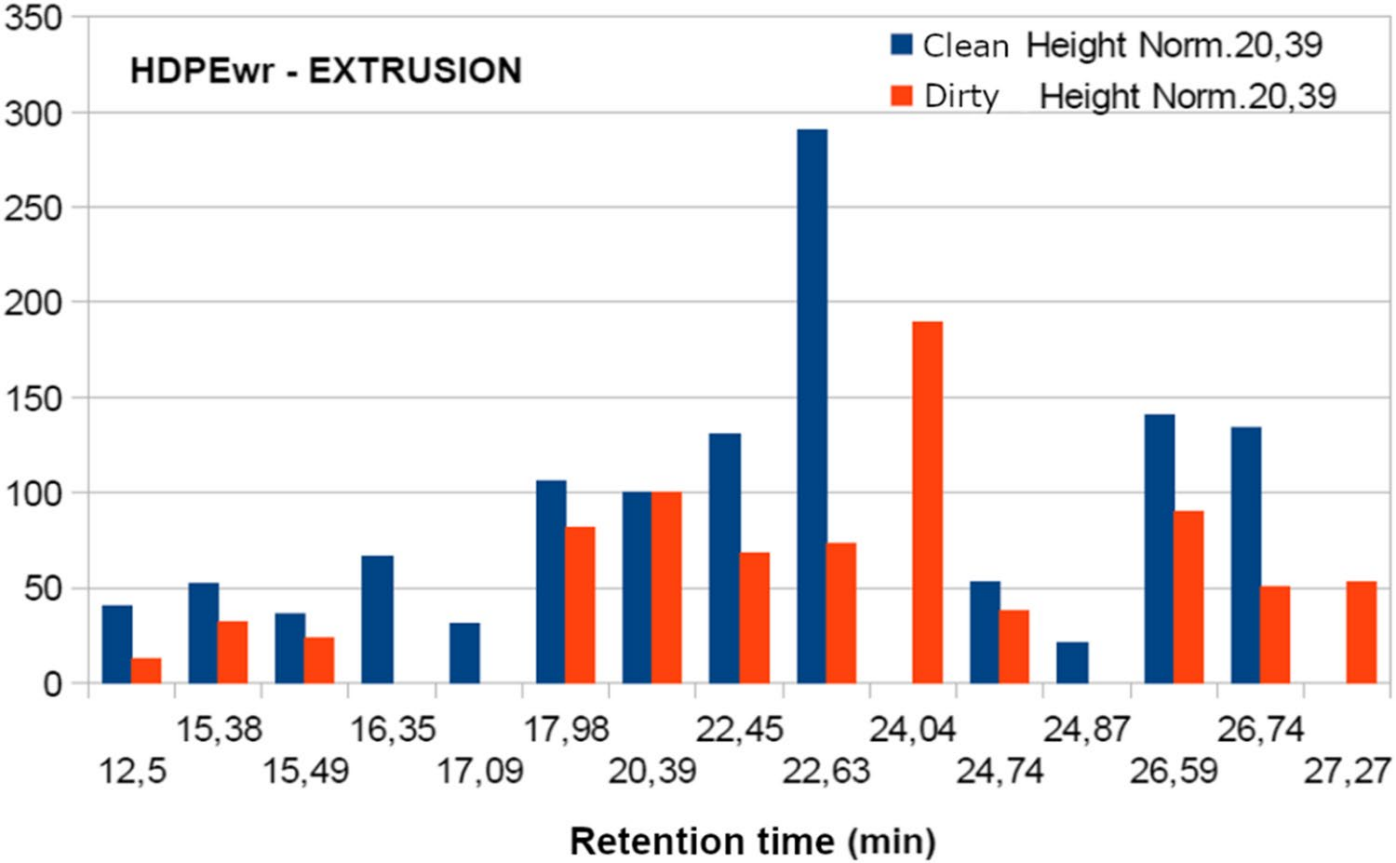
Normalisation of HDPEwr-extrusion peaks from the value 20.39

**Fig. 12:**
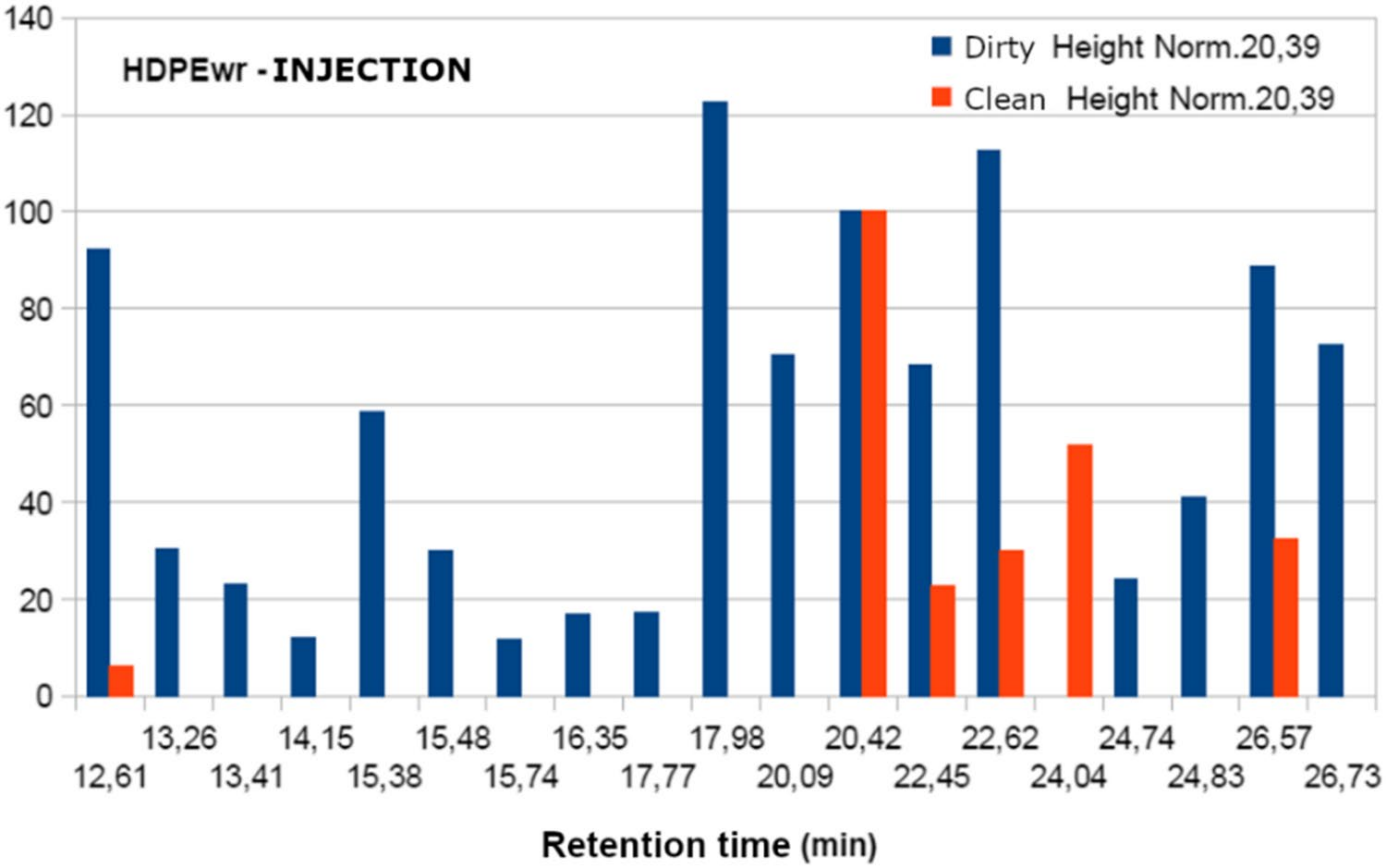
Normalisation of the HDPEwr-injection peaks from the value 20.39

In view of Figs. 10, 11, and 12, it can be observed in the first series without acetone, the decrease in the intensity of the peaks for the HDPEw sample subjected to extrusion and injection, probably due to the thermal treatment of the extrusion and injection processes that favour the loss of volatiles and semivolatiles. Secondly, the acetone-washed series shows a more pronounced and larger decrease in peak intensity compared to the non-acetone-washed samples, which could be due to the ability of acetone to carry away other less volatile compounds.

#### Odour testing by means of olfactory panels

In order to determine the degree of bad smell, a sensory test was used with different volunteers previously selected according to their olfactory aptitude (Vilarrasa García 2014; *Tipos de Plásticos HDPE | Catálogo | ACTECO*, n.d.).

**Fig. 13:**
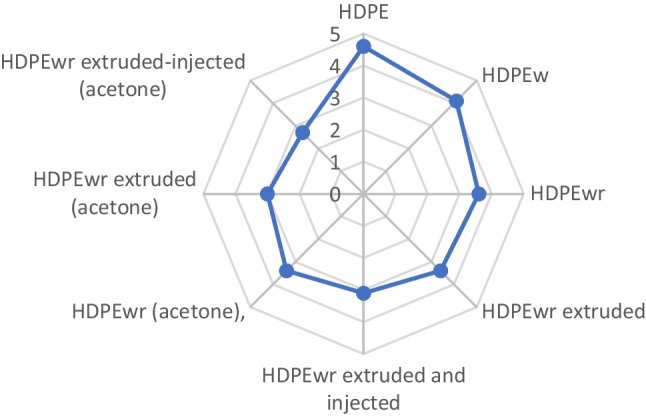
Smell test values

In view of Fig. 13, it has been observed that the washed HDPEw samples compared to the HDPE samples received presented a slight decrease in odour intensity due in principle to the dragging of certain compounds by the chloroform, water and soap of the starting sample. Likewise, the extruded and injected samples compared to the washed HDPEw lose part of this odour, probably due to the fact that the thermal treatments to which the sample is subjected during the extrusion and injection processes favour the elimination of volatiles responsible for the odour. Likewise, HDPEwr, extruded and injected HDPEwr samples treated with acetone reduce the odour much more than the previous samples; this could be due to the effect of the compound acetone widely used as a solvent in the industry to dissolve and clean different materials, greases, oils, plastics, etc.

## Conclusions

The described results show that the starting HDPEwr material has a number of impurities from other polymeric materials analysed by FTIR. Although impurities were detected in the sample, their concentration is relatively low, as DSC observes no melting peaks of other polycrystalline materials. On the other hand, the mechanical characterisation of the HDPEw sample shows similar Charpy tensile strength, elongation and impact energy absorption characteristics to other recycled HDPE. These results show that the different washing and processing treatments do not affect the properties of HDPEw.

Likewise, the pre-washing with acetone of the HDPEwr sample and its subsequent processing eliminates certain volatile compounds and reduces the intensity of the chromatogram peaks of other substances that contribute to the volatile fraction present in these materials compared to the same samples without acetone treatment. In addition to the effect of acetone, the extruded and injected HDPEwr samples lose part of these volatiles, probably due to the fact that the thermal treatments they undergo during processing favour their elimination. Therefore, this type of treatment of HDPE waste can be beneficial for the elimination of volatiles by controlling the temperature during processing and subjecting it to washing treatments with certain organic compounds such as acetone.

## Data Availability

The authors declare that the data supporting the conclusions of this study are available in the paper. If data in another format are required, they can be requested from the corresponding author.
